# Cost-Effectiveness of Home Hemodialysis With Bedside Portable Dialysis Machine "DIMI" in the United Arab Emirates

**DOI:** 10.7759/cureus.18549

**Published:** 2021-10-06

**Authors:** Chandra Mauli Jha

**Affiliations:** 1 Nephrology & Dialysis, Al Mazroui Medical Center, Abu Dhabi, ARE; 2 Nephrology, Nephro Care Home Hemodialysis, Abu Dhabi, ARE

**Keywords:** hemodiafiltration, quality of life on dialysis, home hemodialysis, nurse assisted home hemodialysis, cost effectiveness, portable hemodialysis, chronic kidney disease, end stage renal failure, renal replacement therapies (rrts)

## Abstract

Background and objective

The incidence and prevalence of patients requiring renal replacement therapies (RRTs) are increasing worldwide and a large number of these patients die prematurely due to the unavailability of treatment. While in-center hemodialysis remains the most commonly practiced modality globally, more and more patients find it unsuitable due to their frail condition, difficulty in ambulation, and time lost in traveling, etc. Such patients find the self-administered or nurse-assisted home hemodialysis (NAHHD) more suitable. The costly and recurring nature of these therapies prompted us to evaluate and compare the cost-effectiveness aspect of these two treatment modalities. Thus, the aim of the study was to investigate if home hemodialysis (HHD) with a portable hemodialysis machine was cost-effective in comparison to in-center hemodialysis for patients of end-stage renal failure (ESRF) in the United Arab Emirates (UAE). This is the first study of its kind to be conducted in the UAE.

Methodology

The study topic was developed based on an informal inquiry from the health regulator of Abu Dhabi if HHD was cost-effective compared to in-center hemodialysis with an emphasis on a portable dialysis machine. No such head-to-head study performed in the UAE was available. Hence, a systematic review based on the Preferred Reporting Items for Systematic Reviews and Meta-Analyses (PRISMA) design was chosen as the investigative method. An outline of the study was drafted, and a literature search of Science of Web, PubMed, and Cochrane Evidence was performed using the keywords "Home Hemodialysis", "home-based Dialysis", "Cost-effectiveness of Dialysis", "Cost-effectiveness of renal replacement therapy", etc. A review of the article titles was performed to include the articles relevant to the cost of RRTs and the economic burden of ESRF. Full text and abstracts of those articles were retrieved, studied, and, the articles that were found not relevant were excluded. The remaining articles were studied and used in the evidence synthesis. DIMI was chosen to represent a standard type of recently developed portable dialysis machines.

Results

It was interesting to find out during the review that HHD and in-center hemodialysis had been developed simultaneously but the former had eventually fallen out of favor. The review revealed that HHD is not only as effective as in-center hemodialysis but is also associated with better survival benefits over the latter. Several studies have found it to be significantly cost-effective compared to in-center hemodialysis. Newer types of HHD machines make it easier for the patients or their family/caregivers to administer it safely and effectively at home and while traveling. They have regenerated interest in HHD and the Medicare administration in the USA has already decided to make use of it at a more frequent rate.

Conclusion

Based on the evidence in the available literature, HHD is cost-effective when compared to in-center hemodialysis in terms of survival benefits, quality of life (QoL) of patients, and monetary savings. Newer portable bedside dialysis machines provide better safety and have simplified the procedure of hemodialysis, making HHD more acceptable to patients and caregivers. We believe HHD should be the preferred modality of treatment instead of in-center hemodialysis, and that applies to UAE too.

## Introduction

Kidney disease is defined as an abnormality of the kidney structure or function with huge implications for the health of the affected individual. Chronic kidney disease (CKD) denotes various structural or functional disorders of the kidney present at least for three months, with variability in their clinical presentation, severity, and rate of progression. The concept of CKD was developed based on the recognition that disordered kidney function progresses from less severe to more severe disability at different rates of progression, which if detected early and intervened to slow down the rate of progression, would help in maintaining better health for longer periods for the patients as well as saving expenditure overall [1].

The number of CKD patients is increasing worldwide and CKD is currently the most significant contributor to morbidity and mortality from non-communicable diseases. It is a highly prevalent condition that accounts for a substantial proportion of the disease burden globally. The prevalence of CKD in the global population is about one out of 11 persons (9.1%). Its prevalence has not declined over the past 27 years as compared to the burden of many other important non-communicable diseases [2]. Among patients with CKDs of varying severity, those who suffer from the most severe ones cannot sustain their life and health without a treatment that involves substituting the function of the kidney. Such patients are called end-stage renal disease (ESRD) or end-stage renal failure (ESRF) patients, and, the treatments supporting their life by substituting the function of the kidney are called renal replacement therapies (RRTs). The number of kidney disease patients in general and those who require RRTs are increasing worldwide. Treatment modalities available for ESRD patients are kidney transplants and dialytic therapies. Both modalities are costly therapies, dialytic therapies being costlier than kidney transplants. The number of patients on dialytic treatment is much larger than those undergoing kidney transplants. It is because of the limited number of organs (kidneys) available for transplant and also because many patients are not medically or psychologically fit to undergo transplant surgery. Treatment costs for ESRD patients rose after the 1960s with the advent of dialytic renal replacement techniques, which improved the survival rate of those patients and required the long-term application of those life-saving costly treatments [3]. Dialytic therapies are of two types: peritoneal dialysis (PD) and hemodialysis (HD). Hemodialysis, which is more prevalent worldwide, could be administered either at home or in-center. While different modalities of PD for ESRD patients are carried out at home, hemodialysis at present is largely carried out at hospitals or specialized centers. Dialysis at home can be performed by the patients themselves or family/caregivers, or it could be nurse-assisted. All these forms of dialysis therapies, being costly and recurrent, put a large burden on the health systems globally, which often lead to the unavailability of these methods at times and in some places. Apart from the patient itself, it also affects the life of the family members involved in the care of the patient, leading to a decline in their quality of life (QoL), loss of their employment time, and their earnings. Thus, from both an individual and societal standpoint, even a small reduction in the cost of these services may have a marked impact in the long run.

Liyanage et al. found in their most liberal estimate that among the 2.6 million patients who received RRTs worldwide in the year 2010, only around 50% had actually required it. They observed a shortage of renal replacement services in many countries, resulting in the possible premature death of around 2.28 million adults from a lack of access to this treatment in 2010. They projected that the requirement would double by the year 2030. By their estimate, the number of patients receiving RRT worldwide in 2021 would be approaching 3.8 million [4]. On a similar note, Anand et al. estimated in their study that in 2010, there were at least 1.2 million premature deaths among diabetes and elevated blood pressure patients due to a lack of access to RRT and as many as 3.2 million premature deaths due to all causes of ESRD [5].

The current prevalence of CKD in the USA is around 15% with ESRD cases increasing at an average of 2.5% annually since 1996 [6]. This increase in the incidence and prevalence of ESRD patients is complicated by the increasing proportions of elderly patients and patients with multiple comorbidities among them. Currently, about half of the ESRD patients have diabetes and a majority of them have cardiovascular diseases [7]. An increasing proportion of elderly patients, frail patients, patients with diabetes, and patients with complex coexisting conditions, many of them not fully ambulatory and find the frequent travel to the dialysis center difficult, have been using hemodialysis. Such patients are less capable of self-care and unable to perform complicated procedures like dialysis. For such patients "nurse-assisted home dialysis program" is a very promising alternative [8].

Home hemodialysis (HHD), among the different types of RRTs, is not a new concept. Charles Kirby, a cardiac surgeon, in his presidential address to the American Society for Artificial Internal Organs (ASAIO) in 1961 talked about HHD as follows: "Perhaps what we need is a home dialysis unit to be placed by the patient's bedside so that he can plug himself in for eight hours once or twice a week" [9].

If we examine the history of the home and in-center dialysis, we find that both had been developed almost simultaneously as per the requirement of patient care. The technology of dialysis for saving the life of kidney failure patients was introduced for community use in 1962 when the Seattle Artificial Kidney Center was set up by Scribner and James Haviland [10]. It was followed in 1963 by the development of a miniature single-patient version of the machine by Babb, a professor of nuclear engineering at the University of Washington, and his team, which was intended for unattended HHD for a young girl patient named Caroline. Subsequently, it began to be used for HHD. Based on the experience during those years, thrice-weekly dialysis was established as a widely practiced and accepted standard [11,12,13,14,15].

As early as 1965, Hampers and Merrill from Boston reported about the successful use of HHD in four young male patients for more than a year in the Annals of Internal Medicine. They reported that the patients had welcomed the sense of independence associated with HHD, and they had achieved a full work week because the dialysis was usually done in the evenings; moreover, they felt that they were participating in their care and hence had some control over their future. The flexibility of the dialysis schedule to suit the individual's social, business, or medical needs was a great advantage over the rigid schedule of any hospital program [16].

In the USA, when the Medicare Act provided people with coverage for RRT in 1972, 40% of patients were undergoing HHD, which declined to only 0.7% by 2003 [17]. In his review article, CR Blagg has discussed the reasons for the change in the practice trend and stressed why HHD should be the preferred treatment of choice [18]. As per the estimates of Medicare stakeholders in 2016, 50% of ESRD patients in the USA could be eligible for HHD while the utilization was merely around 4%. Medicare has set a target of 25% utilization of HHD for ESRD patients, which has not been attained yet [19].

**Figure 1:**
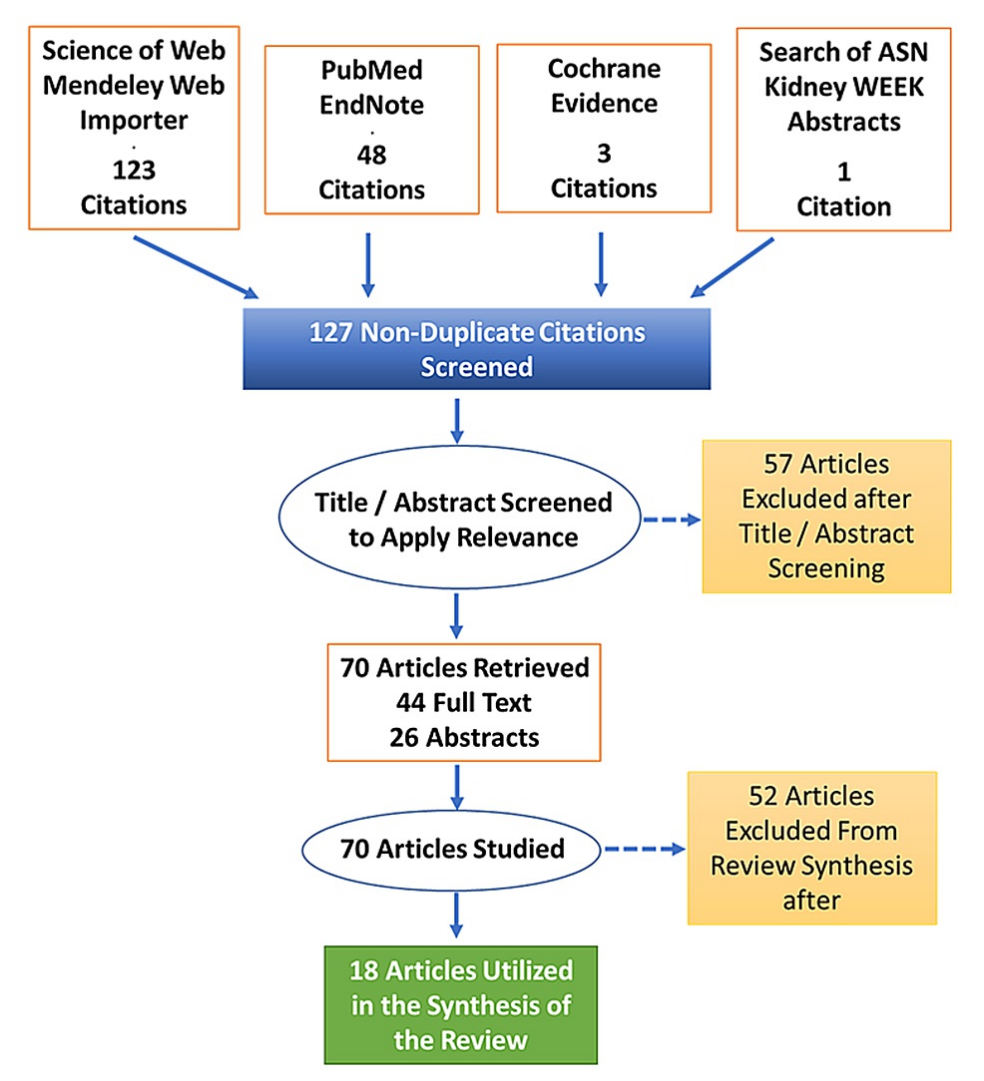
Prisma flow diagram PRISMA: Preferred Reporting Items for Systematic Reviews and Meta-Analyses

HHD as a treatment practice for kidney failure patients is useful and superior in many ways but is currently the least practiced method. There has been a renewed interest in the HHD among different stakeholders involved in the care of patients requiring RRTs. In light of this renewed interest in HHD and the high cost of RRTs, an economic evaluation of this modality of therapy compared to the other modalities is required. The purpose of this study was to explore the utility of this treatment modality in the United Arab Emirates (UAE) in terms of cost-effectiveness. The research question was developed based on an inquiry from the health regulator of Abu Dhabi if home dialysis with a portable dialysis machine was cost-effective compared to in-center hemodialysis. The machine "DIMI" was chosen as a representative of several portable hemodialysis machines that have been developed recently and approved by health regulators of the USA and the European Council (EC). The methodology of the study is illustrated in the Preferred Reporting Items for Systematic Reviews and Meta-Analyses (PRISMA) diagram below (Figure 1).

## Materials and methods

The research question developed was as follows: "Would HHD using a portable hemodialysis machine compared to in-center hemodialysis be cost-effective in the UAE?" This question was developed to find the evidence-based answer for a similar informal inquiry from the health authority of Abu Dhabi (UAE). The machine DIMI was selected as a representative of the standard type of newly developed portable bedside hemodialysis machine, which had the advantage over other machines to deliver all modalities of hemodialytic therapies including hemodiafiltration.

Since no similar studies had been performed in the past among patients in the UAE, to answer the study question, the PRISMA model of a systematic review of published literature was planned. A literature search of PubMed, Science of Web, and Cochrane Review databases was performed using the keywords "Home Hemodialysis", "Home-based Dialysis", "Cost-effectiveness of Dialysis", "Cost-effectiveness of renal replacement therapy", "cost of hemodialysis" etc. The literature search was restricted to the period from 1st January 1960 to 31st January 2021. Title and abstract screening of 127 non-duplicate citations were performed to apply the criteria for relevance; 57 citations that were not related to hemodialysis, those related to dialysis but not related to chronic hemodialysis, and those related to acute kidney Injury only were excluded. Forty-four full text and 26 abstracts among the 70 citations were selected and studied, and 18 articles out of those 70 were found to be contributory toward evidence synthesis in quantitative and qualitative terms (Figure 1). Apart from an analysis of the selected articles, we also engaged in a thorough review of the literature on the topic [1-52].

Nine studies provided evidence that HHD was effective or superior to in-center hemodialysis in terms of survival benefits. There was only one study done in that UAE, which was not comparative but had analyzed the QOL issues of patients who had received NxStage-based nurse-assisted HHD (NAHHD). Eight papers that consisted of reviews, analyses, or studies had addressed the cost-effectiveness of HHD as compared to in-center hemodialysis and/or other modalities of RRTs (Table 1).

**Table 1:** Studies comparing home hemodialysis to other modalities of renal replacement therapies *All forms of HHD (conventional, long, frequent, or long/frequent sessions) vs. all forms of PDs (continuous ambulatory or automated PDs); **17% at 28 years HHD: home hemodialysis; PD: peritoneal dialysis

Studies					
Effectiveness studies					
Author	Type of study	Duration/period	Features of study	Result of study	Country
Hampers and Merrill [16]	HHD/ICD study	13 months	4 patients, twice weekly	Advantageous	USA
Weinhandl et al. [24]	HHD/ICD study	2005-2007	1,873 HHD/9,365 in-center dialysis. Daily vs. 3 per week	Lower risk of death for HHD patients	USA
Nadeau-Fredette et al. [25]	HHD/PD study	2000-2012	706 HHD/10,710 PD	Better patient survival on HHD	New Zealand/Australia*
Mailloux et al. [26]	HHD/ICD/PD study	1970-1993	74 HHD/896 ICD	Better patient survival on HHD	USA
Delano [27]	HHD study	1969 + 28 years	204 patients	Long-term technique survival**	USA
Arkouche et al. [28]	HHD study	1974-1997	471 patients	Better patient survival on home t/t	France
Covic et al. [29]	HHD/ICD/PD study	1968-1986	54 HHD/286 total	Better long-term survival on HHD	UK
McGregor et al. [30]	HHD study	1969-1998	334 HHD	Better patient survival on HHD	Australia/New Zealand
Woods et al. [31]	HHD study	1986-1987	70 HHD/3,102 ICD	Better patient survival on HHD	USA
Bernieh et al. [48]	HHD study	1 year	QoL of nurse-assisted HHD patients using NxStage system in the UAE	Nurse-assisted HHD with NxStage system in the UAE has good QoL, efficacy, and satisfaction for patients and their families	UAE
Cost-effectiveness studies					
Author	Type of study	Duration/period	Features of study	Result of study	Country
Walker et al. [34]	Review	2000-2014	6 studies/231 articles	Contemporary HHDs are less costly and more effective than facility HD	
Klarman et al. [35]	Analysis	41 (dialysis) and 28 (Tx) years	Cost-effectiveness analysis of RRTs	US$7,400 difference in the cost of per life-year gained in favor of HHD as compared to in-center HD	USA
Lee et al. [36]	Study	1999-2000	Prospective cost analysis of HHD/ICD	Overall annual cost of care for HHD was cheaper than that for in-center dialysis	Canada
Mowatt et al. [38]	Review			HHD less expensive than hospital IHD	UK
Croxson and Ashton [39]	Analysis		Cost-effectiveness analysis of HHD/ICD/CAPD	HHD cheaper than ICD	New Zealand
Krahn et al. [40]	HHD/ICD/PD study	2006-2014	9-year period, 12,691 adult patients	Starting HHD cheaper than facility dialysis	Canada
Howard et al. [41]	HHD/ICD/PD study	2005-2010	Modeling based on registry data	Switching new patients from ICD to HHD saves money	Australia

## Results

Is home hemodialysis effective?

The effectiveness of HHD has to be established before its cost-effectiveness with in-center hemodialysis can be compared. The effectiveness of HHD can be examined by different parameters such as survival and mortality, QoL, cardiovascular outcomes, effect on mineral bone disease, and side effects, etc. For brevity, we restricted this study to survival and mortality, and, QoL, which also encompassed the cardiovascular component. 

Whenever survival benefit among dialysis patients of different modalities has to be compared, the dose of dialysis delivered has to be considered so that the outcome is measured on an equivalent basis. The famous HEMO study published in 1982 looked into the effects of the dose of dialysis and the level of the flux of the dialyzer membrane on mortality and morbidity among patients undergoing maintenance hemodialysis. The dosage defined the quantity of dialysis while the flux of the membrane decided the size of the molecules that could be removed during dialysis; a higher flux membrane could remove larger molecules. The randomized HEMO study involving 1,846 patients undergoing hemodialysis thrice weekly found that there was no major benefit from a higher dialysis dose than that recommended by contemporary US guidelines, which entailed the urea-reduction ratio of 66.3 ±2.5%, the single-pool Kt/V 1.32 ±0.09, and the equilibrated Kt/V 1.16 ±0.08, or the use of a high-flux membrane [20].

Because the same dose of dialysis could be delivered over variable durations, the effect of the duration of dialysis in addition to dialysis dose was studied by various researchers later on. Different studies have confirmed that dialysis duration of fewer than four hours a session three times a week was associated with an increased mortality rate of up to 42%. Longer duration of dialysis with same dialysis dose was associated with improved cardiac status and chance of survival benefit [21,22,23].

Having set the dosage of dialysis, the survival benefit could be compared between different modalities of hemodialysis. Several studies have compared survival benefits between in-center hemodialysis and HHD. Weinhandl et al. compared mortality in HHD patients who initiated RRT with the NxStage System One (similar to DIMI) from 2005 to 2007 with matched thrice-weekly in-center hemodialysis patients. The study found a weak beneficial effect of HHD on the risk of death. They found that the risk of death for daily HHD patients was 13% and 18% lower in intention-to-treat and as-treated analyses, respectively [24]. Nadeau-Fredette et al. found that HHD was associated with better patient survival than treatment with PD (five-year survival: 85% vs. 44%, respectively; log-rank: p<0.001) [25]. This study showed excellent survival results. Several other studies have found that HHD provided the best patient survival rates [26,27,28,29,30]. Woods et al. used data from the United States Renal Data System (USRDS) and found that the unadjusted relative risk of death among HHD patients compared with center dialysis patients was 0.37 (p<0.01). If controlled for age, race, gender, and cause of renal failure, the relative risk was 0.58 (p=0.02), and with additional adjustment for comorbid conditions, it was 0.57 (p=0.03) [31]. These studies support the hypothesis that HHD is not only effective but it also has significant survival benefits over the in-center dialysis.

Is home hemodialysis cost-effective?

RRT is one of the costliest therapies. Approximately 1% of the health budget is accounted for by patients of dialysis and transplant. Even a small change in cost per procedure would result in a huge change in the cost borne by the system.

An economic evaluation of the treatment is a difficult subject in which a comparative analysis of the alternative courses of action is performed. It deals with both the inputs and outputs, which can be described as the costs and consequences of alternative courses of action. It can be done as cost-effectiveness analyses (CEAs) where a single common effect that may differ in magnitude between the alternative programs is compared. A variant of cost-effectiveness is cost-utility analysis in which, for the consequences, a generic measure of health gain such as quality-adjusted life-year (QALY) is measured. A cost-benefit analysis estimates the additional health benefits of a given intervention and the additional cost benefits associated with achieving those health benefits [32,33].

The outcomes of cost-effectiveness evaluation are presented as the "incremental cost-effectiveness ratio" (ICER), a ratio of the difference in costs between two interventions, divided by the difference in their respective outcomes [34]. Several researchers have tried to explore the economics of the RRTs. Most of them have reported their findings in terms of cost-saving.

Walker et al. performed a systemic review of the cost-effectiveness of contemporary HHD modalities compared with facility hemodialysis. They concluded that HHD modalities including nocturnal and daily HHD were cost-effective or cost-saving compared with facility-based hemodialysis because of lower staff costs, and better health outcomes for survival and QoL. They observed that expanding the proportion of hemodialysis patients managed at home was likely to produce cost savings [34].

As early as 1968, Klarman et al. noted a $7,400 difference in the cost per life-year gained in favor of HHD as compared to conventional hemodialysis. It was $11,600 vs. $4,200 for conventional hemodialysis and HHD, respectively. The cost-effectiveness ratio (the difference in cost of HHD and conventional hemodialysis divided by the difference in their effect) was markedly in favor of HHD compared to conventional hemodialysis [35]. An annual cost of care difference of $21,000 ($51,252 for in-center hemodialysis vs. $29,961 for HHD; p<0.001) was noted by Lee et al. [36].

Ashton and Marshall explored the organization and financing of dialysis and kidney transplantation services in New Zealand. They noted that in New Zealand, there was optimum utilization of home dialysis compared to in-center dialysis. In New Zealand, 41% of patients were treated at hemodialysis units while 59% were treated at home: 45% home PD and 14% HHD (ANZDATA). Estimated costs (NZ$) for ESRD modalities in New Zealand during 2002-2004 were as follows: hospital hemodialysis: NZ$64,318 per patient per year; HHD: NZ$33,548 per patient per year. Most likely, the funding constraints encouraged the physicians and patients to choose higher utilization of HHD therapies, which kept the total expenditure per ESRD patient relatively low [37].

Mowatt et al. performed a comprehensive systemic review of effectiveness and cost-effectiveness, and economic evaluation of HHD vs. hospital or satellite unit hemodialysis for people with ESRF and found that HHD was less expensive than hospital hemodialysis. In their view, with an increasing number of ESRD patients, a corresponding increase in HHD offered an option for restricting increases in the RRT budget [38].

Croxson and Ashton performed an economic evaluation of continuous ambulatory peritoneal dialysis (CAPD), HHD, in-center hemodialysis, and transplantation using cost-effectiveness analysis to evaluate the cost per life-year saved. They noted that the value of the cost per life-year saved, expressed in 1988 $NZ, was 35,270 for in-center dialysis, 28,175 for HHD, and 26,390 for CAPD [39].

Krahn et al., from the Toronto Health Economics and Technology Assessment Collaborative, used the Canadian Organ Replacement Register (CORR) to study 15,240 patients aged 18-105 years who initiated chronic dialysis over a period of nine years between 1st April 2006 to 31 March 2014 in the Canadian province of Ontario, to evaluate the costs and the survival data. The highest five-year unadjusted survival was for HHD patients (80%), followed by PD (52%), and it was lowest for facility hemodialysis (42%). The mean 30-day cost (as-treated) for patients receiving HHD was 64% lower than for facility hemodialysis patients [40].

In their study, Howard et al. used a multiple cohort Markov model to assess costs and health outcomes of RRT for new ESRD patients in Australia during 2005-2010. They concluded that switching new patients from hospital hemodialysis to HHD could save A$46.6 million by 2010 [41]. de Wit et al. studied the cost-effectiveness and cost-utility of dialysis and transplantation over a period of five years by using a Markov-chain model based on the actual Dutch ESRD program and found in-center hemodialysis to be the least cost-effective treatment. They concluded that in countries where in-center hemodialysis was the only or the major treatment option for ESRD patients, substitutive policies for home-based treatment like HHD or CAPD would have a substantial impact on the cost-effectiveness of ESRD treatment [42].

## Discussion

The number of CKD patients and ESRD patients is increasing globally. Kidney transplant has several limitations, especially that of availability, which will result in an increasing number of ESRD patients on different dialytic treatments including hemodialysis. Despite the findings that HHD is associated with the best patient survival rates, a better quality of life, better chances of rehabilitation and ability to work, better control of blood pressure, etc., its use has declined gradually over time. In fact, this trend seems quite unreasonable. In the USA, this was partly due to inadequate payments for HHD modality for the first five years of the Medicare system. It should be noteworthy that the practice pattern in the USA is considered a standard model to be followed in many other countries. Another reason for the decline was that many patients were considered unfit for self-care either by the physician or by the patients themselves. Once a patient attends in-center dialysis, he or she is likely to develop "learned helplessness" [43]. This learned-helplessness makes the patient depend more on hospitals and clinics, while ideally, the patients of chronic diseases like ESRD should have been involved in their own care [44]. To develop and maintain this required self-care, training and involvement from treating physicians and nurses to impart education are required. That requires a well-structured program. A successful HHD program is more than just a treatment modality. In fact, it is more a system than a treatment [45]. At present, the interest in HHD has re-emerged among different stockholders including the industry. As mentioned above, the Medicare administration in the USA in 2016 has estimated that 50% of ESRD patients in the USA could be eligible for home dialysis. Medicare plans to increase the acceptance of HHD and has set a target of 25% utilization of HHD for ESRD patients [19].

The dialysis medical industry has been actively engaged since 1991 to develop new hemodialysis machines more suitable for use at home in terms of portability and lesser requirement of space, simplification, and improved safety. Different hemodialysis machines developed and approved by health authorities for use as "home hemodialysis machines" are (1) The Baxter VIVIA Hemodialysis System (Baxter Healthcare Corporation, Deerfield, IL), (2) Fresenius Medical Care 2008K@Home Dialysis Machine (Fresenius Medical Care AG & Co., Bad Homburg, Germany), (3) NxStage System One (NxStage Medical, Inc., Lawrence, MA), (4) Quanta SelfCare+ (Quanta Dialysis Technologies, Alcester, UK), (5) Physidia S³ device (Physidia Medical Devices, Saint-Barthélemy-d'Anjou, France), and (6) DIMI, etc. [17].

The Baxter VIVIA hemodialysis system was designed to deliver high-dose hemodialysis, which could provide all types of hemodialysis at home. It provided reuse of the dialyzer and bloodlines employing heat disinfection and automatic prime and rinse-back. An integrated access disconnect system; an animated, patient-friendly, graphic user interface; wireless connectivity to the clinic; an integrated heparin pump; an integrated water treatment source; and online dialysate generation were the other cutting-edge technologies available with that system. The drawback was that it was not portable, and it lacked an integrated blood pressure monitor system. It was approved by EC in 2013 but unfortunately, Baxter withdrew it in 2016 [46]. The 2008K@Home machine by Fresenius was similarly withdrawn. It could also provide all types of hemodialysis. In addition to other features of VIVIA, it had an integrated blood pressure monitor, and dialysate concentration could also be varied. It lacked a reuse system and required larger space. It had "WetAlert", a wireless wetness monitor at the needle site, which would stop the blood pump if the alarm was activated. It had some drawbacks as well: the machine was not portable, required an external water treatment source, larger space, significant home remodeling, and higher initial setup cost. Dissimilar to these two machines and more similar to PD Cycler are the other machines: NxStage System One, Quanta SelfCare+, Physidia S³, and DIMI. These four machines are portable. These can be used during travel, do not require large space except for the storage of consumables. These have battery backup in case of power shutdown. These are similar in their function and simplify the connection of the patient to the machine. These machines use a disposable drop-in cartridge with blood and dialysate lines with a dialyzer attached. The last one in this group, DIMI, can be used to carry out PD or other diafiltration treatments like hemodiafiltration too.

As mentioned above, these machines use disposable drop-in cartridges with blood and dialysate lines with a dialyzer attached. NxStage System One has been used in the USA and UAE. In their report about their one-year experience with NAHHD by using the NxStage machine in bed for homebound and multi comorbid hemodialysis patients, Bernieh and Calaud from UAE confirmed its efficacy, good quality, and safety. It had a significant positive impact on the QoL and satisfaction of both patients and their families [47,48]. Komenda et al. has reported on the successful use of QuantaC+ [49]. DIMI has been reported to provide promising results by Di Liberato et al. [50].

HHD is both a treatment modality as well as a system. In the UAE, HHD has been provided since 2016. At present, there are two active service providers of home hemodialysis: NMC Provita International Medical Centre and Home Hemo Dialysis. Both service providers provide NAHHD. NMC Provita uses regular hemodialysis machines fixed at patients' homes while Home Hemo Dialysis uses NxStage System One. Efficacy of this system using a portable dialysis machine, e.g., DIMI in UAE, has already been reported [48]. There is no official exact estimate of the number of patients requiring RRT in the UAE. But observes in the field have suggested that the number is on the rise. SEHA Kidney Care, the largest provider of RRTs in Abu Dhabi, has reported that the total delivered hemodialysis sessions were over 77,314 on a thrice-weekly basis from the beginning of March till the end of August 2020 [51]. Based on this, we can estimate that a total of 1,074 patients were dialyzed regularly by SEHA Kidney Care. In addition, there are other providers like NMC Royal Hospital, Mediclininc Hospital, Ahalia Hospital, Burjeel Hospital, and Al Mazroui Medical Centre Day Surgery, etc. which are also providing hemodialysis. If we assume that the services provided by these operators amount to at least one-fifth of those by SEHA Kidney Care, the total number of patients would be around 1,300, which would amount to 590 patients per million people.

Since 2020-21, the world has been facing a severe healthcare crisis in the form of the coronavirus disease 2019 (COVID-19) pandemic, and HHD can provide increased benefits for dialysis patients in this situation.

Because the cost-effectiveness can be derived by parallelism, the tested effectiveness of NxStage System One would be applied to DIMI. DIMI-based therapy cannot be inferior or less cost-effective than NxStage System One-based therapies. Since the payment to the service provider is not differentiated based on the device used but by the service, and HHD is a cost-effective service with greater survival and QoL benefits, dialysis service rendered at home would be cost-effective. It would save (1) the expenditure on the part of the patient's family over traveling, and (2) the loss of work for relatives who would be required to assist the patients three times every week. In such a situation, the part of the earnings at the service provider end is transferred indirectly to the patients and their families who turn out to be the real beneficiaries. Compared to NxStage System One, DIMI can provide improved quality and cost-effectiveness in dialytic treatment because this machine can also carry out hemodiafiltration, which has been proven to be more advantageous than simple hemodialysis [52].

## Conclusions

Based on the discussion above, it could be confirmed that any HHD service including that with DIMI would be cost-effective anywhere, including the UAE. NAHHD, which is the currently available service model of HHD, provides multiple benefits to the patients and their caregivers, and it should be actively promoted. The limitation of this study is that it derives its conclusion from parallelism since there is no head-to-head study available on the cost-effectiveness among the UAE dialysis population comparing HHD to in-center dialysis. A study in the future addressing this aspect would be interesting and would also provide more accurate data. There is a lack of data regarding the demographic characteristics of the on-dialysis population in the UAE. Hence, we do not know if the study population in the studies we included in our review were similar to the UAE dialysis population or not. Thus, this study also provides awareness about this information gap as well as possible areas of required research on this subject.
